# Editorial: Aging and frailty: from causes to prevention

**DOI:** 10.3389/fragi.2025.1723871

**Published:** 2025-11-06

**Authors:** Emiliana Giacomello, Luana Toniolo

**Affiliations:** 1 Department of Medicine, Surgery and Health Sciences, University of Trieste, Trieste, Italy; 2 Laboratory of Muscle Biophysics, Department of Biomedical Sciences, University of Padova, Padova, Italy

**Keywords:** aging, frailty, lifespan, healthspan, longevity

## Introduction

Recent improvement in sanitary, nutritional, and socioeconomic conditions has led to an increase in life expectancy. As a result, by 2050, people aged more than 60 years are expected to double, and people aged more than 80 years are expected to triple (https://www.who.int/news-room/fact-sheets/detail/ageing-and-health). Unfortunately, increased lifespan, is not always paralleled by an adequate healthspan, because of interference of social, behavioral, physiological, cellular, molecular, and less known factors (Balcombe and Sinclair, 2001). This complexity makes difficult the definition of aging, and challenging management of this period of life.

Aging is accompanied by decline of the individual resulting in a complex condition called frailty, characterized by loss of physical and psychological abilities, and by an amplified vulnerability to stress factors (Morley et al., 2013; Hoogendijk et al., 2019). To limit the impact of frailty on wellbeing and on society, to improve both preventive actions and management, a deep understanding of this condition is needed.

Considering frailty as a geriatric syndrome, we need to understand its etiology, define diagnostic parameters, and apply therapeutic and prevention approaches. This Research Topic was aimed at collecting new knowledge on different aspects of frailty. It comprises 13 research articles that provide new information in different aspects of aging and frailty, such as comprehension of molecular determinants and socioeconomic influence, frailty definition and biomarkers, impact on health outcomes, and prevention strategies.

## Biological basis of frailty

Frailty is the result of a multisystem derangement that involves metabolic unbalance, systemic inflammation, musculoskeletal malfunctioning and altered responses to stress (Picca et al., 2022). Among the multiple biological variables involved in aging and muscle weakness (Picca et al., 2022; Li et al., 2024; Sato et al., 2024), the genetic background captures much interest (Baghdadi et al., 2022; Sirago et al., 2022). Interestingly, Krasniqi et al. report on the impact of genetic variants of vitamin D receptor on muscular fitness in middle aged and older adults. This evidence confirms the importance of biological mechanisms and calls for further analysis of genetic predisposition to develop individual strategies.

## Socioeconomic influence on frailty

Recent data evidence that biological variables are not the only determinants of frailty, on the contrary, they highlight the role of variables related to the socioeconomic status of individuals, stimulating the interest to analyze the correlation among frailty and socioeconomic conditions. Accordingly, in the present topic several articles investigate this important theme. The Chinese Longitudinal Healthy Longevity Survey (CLHLS 2008–2018) reveals that higher levels of frailty and lower levels of social participation exhibit significant bidirectional relationships with age, education level, marital status, and drinking habits (Bi et al.). Analogously, Rahman et al., suggest that health outcomes following traumatic brain injury in Bangladesh depend on socioeconomic settings, since lower status individuals have limited access to treatments. Moreover, as highlighted by Czyżewski et al., geriatric patients, lacking their independence, need special care, that can be provided depending on their location. Their data propose that the number of accesses to emergency from rural areas-based patients prevail to those from the urban areas, maybe due to limited access to a primary care physician.

## Frailty definition and biomarkers

Frailty is a dynamic condition that can appear with physiological, psychological signs or both, making difficult the evaluation of risk factors and formulation of a diagnosis (Morley et al., 2013). The multifactorial nature, and the diverse clinical manifestations limit the establishment of a unequivocal frailty score (Rockwood et al., 2007). According to above reported observations, the evaluation of frailty risk should include both biomarkers and socioeconomic parameters. From the physiological point of view, frailty is characterized by a low-grade chronic inflammation. Therefore, inflammatory markers seem good candidates to help to define and diagnose frailty. In this context, Zhang et al., based on data from the United States National Health and Nutrition Examination Survey (NHANES, 2007–2018), suggest that Systemic Immune-Inflammation Index (SII) and Systemic Inflammatory Response Index (SIRI) could be used as markers of frailty. From the analysis of 16,705 middle-aged and older participants to NHANES 1999–2018, Tang et al. found six complete blood count-derived inflammatory markers (neutrophil-to-lymphocyte ratio, monocyte-to-lymphocyte ratio, platelet-to-lymphocyte ratio, SII, SIRI, and pan-immune inflammation value), which are associated with higher risk of frailty and mortality. Since frailty is also accompanied by a sensible reduction of skeletal muscle strength and endurance, Fujikawa et al. suggest bimanual coordinated movements analyses to assess levels of frailty. From a study on 358 community-dwelling older adults, they found that frail adults exhibit less movement during bimanual coordination tasks compared with non-frail adults. Interestingly, Lin et al., who aimed at assessing the risk of frailty in older adults affected by atrial fibrillation, suggest a comprehensive predictive model based on multiple risk factors, such as age, gender, history of coronary heart disease, number of chronic conditions, sleep quality, and mental health condition. In agreement with the multifactorial nature of frailty, a comprehensive method could be helpful in obtaining a broader picture of frail individuals and fundamental to frailty management.

## Therapeutic, prevention and care approaches

In clinical situations, frail individuals could require different care and may have different outcomes to health challenges, therefore assessment of frailty may provide important decision-making information. In this context, the article from Adamuz et al. reports that COVID-19 patients older than 75 years presented more care complexity individual factors (CCIFs), especially those related to comorbidity, cognitive and social impairment, than younger subjects. On the other side, Ma et al. suggest that frailty is a good parameter to predict postoperative disability after cardiac surgery. Lastly, from their investigation on impact of age and frailty on key clinical outcomes from liver transplants, Valenti et al., found that frailty, rather than age, is a predictor of mortality. Considered the social impact of frailty in several aspects of daily life, there is a growing interest on both healthcare system and community interventions that could reduce the effects of frailty. A study from Ni et al., emphasize the consequence of different activities on frailty of older adults in China. Actually, from the China Health and Retirement Longitudinal Study (CHARLS 2020), it emerges that physical, social, economic, information and sleep activity have positive effects on frailty. Interestingly, a case study reporting a community-based early frailty intervention program delivered by trained laypersons in Singapore, indicates that in an initial phase older adults can be supported by non-healthcare professionals that control physical activity and nutrition of participants (Jayaprakash et al.). Nevertheless, although programs managed by trained laypersons could be an initial step towards awareness of frailty and an initial action towards its combat, these programs need supports and resources, and involvement of healthcare systems (Cesari et al., 2016).

Frailty is a complex multifactorial condition that can accompany aging with multiple pathophysiological manifestations, making each frail individual rather unique. Articles included in this topic provide new knowledge on frailty, confirm the role of biological variables and highlight the weight of socioeconomic factors, that should be considered in the evaluation of risks, diagnosis and therapeutical approaches. Hopefully, new knowledge will increase awareness of frailty, and involvement of society and healthcare systems to promote prevention and care strategies (Cesari et al., 2016).

## References

[B1] Baghdadi M. Hinterding H. M. Partridge L. Deelen J. (2022). From mutation to mechanism: deciphering the molecular function of genetic variants linked to human ageing. Briefings Funct. Genomics 21, 13–23. 10.1093/bfgp/elab005 33690799 PMC8789301

[B2] Balcombe N. R. Sinclair A. (2001). Ageing: definitions, mechanisms and the magnitude of the problem. Best Pract. and Res. Clin. Gastroenterology 15, 835–849. 10.1053/bega.2001.0244 11866480

[B3] Cesari M. Prince M. Thiyagarajan J. A. De Carvalho I. A. Bernabei R. Chan P. (2016). Frailty: an emerging public health priority. J. Am. Med. Dir. Assoc. 17, 188–192. 10.1016/j.jamda.2015.12.016 26805753

[B4] Hoogendijk E. O. Afilalo J. Ensrud K. E. Kowal P. Onder G. Fried L. P. (2019). Frailty: implications for clinical practice and public health. Lancet 394, 1365–1375. 10.1016/S0140-6736(19)31786-6 31609228

[B5] Li Y. Tian X. Luo J. Bao T. Wang S. Wu X. (2024). Molecular mechanisms of aging and anti-aging strategies. Cell Commun. Signal. 22, 285. 10.1186/s12964-024-01663-1 38790068 PMC11118732

[B6] Morley J. E. Vellas B. Abellan van Kan G. Anker S. D. Bauer J. M. Bernabei R. (2013). Frailty consensus: a call to action. J. Am. Med. Dir. Assoc. 14, 392–397. 10.1016/j.jamda.2013.03.022 23764209 PMC4084863

[B7] Picca A. Calvani R. Marzetti E. (2022). Multisystem derangements in frailty and sarcopenia: a source for biomarker discovery. Curr. Opin. Clin. Nutr. and Metabolic Care 25, 173–177. 10.1097/MCO.0000000000000828 35238804

[B8] Rockwood K. Andrew M. Mitnitski A. (2007). A comparison of two approaches to measuring frailty in elderly people. J. Gerontol. A Biol. Sci. Med. Sci. 62, 738–743. 10.1093/gerona/62.7.738 17634321

[B9] Sato R. Vatic M. Peixoto da Fonseca G. W. Anker S. D. von Haehling S. (2024). Biological basis and treatment of frailty and sarcopenia. Cardiovasc Res. 120, 982–998. 10.1093/cvr/cvae073 38828887

[B10] Sirago G. Picca A. Giacomello E. Marzetti E. Toniolo L. (2022). The contribution of genetics to muscle disuse, retraining, and aging. Genes (Basel) 13, 1378. 10.3390/genes13081378 36011290 PMC9407110

